# Natural Melanogenesis Inhibitor, Antioxidant, and Collagen Biosynthesis Stimulator of Phytochemicals in Rice Bran and Husk Extracts from Purple Glutinous Rice (*Oryza sativa* L. cv. Pieisu 1 CMU) for Cosmetic Application

**DOI:** 10.3390/plants12040970

**Published:** 2023-02-20

**Authors:** Pichchapa Linsaenkart, Warintorn Ruksiriwanich, Pensak Jantrawut, Chuda Chittasupho, Pornchai Rachtanapun, Kittisak Jantanasakulwong, Sarana Rose Sommano, Chanakan Prom-u-thai, Sansanee Jamjod, Chaiwat Arjin, Korawan Sringarm, Francisco J. Barba

**Affiliations:** 1 Doctor of Philosophy Program in Pharmacy, Department of Pharmaceutical Sciences, Faculty of Pharmacy, Chiang Mai University, Chiang Mai 50200, Thailand; 2 Department of Pharmaceutical Sciences, Faculty of Pharmacy, Chiang Mai University, Chiang Mai 50200, Thailand; 3 Cluster of Research and Development of Pharmaceutical and Natural Products Innovation for Human or Animal, Chiang Mai University, Chiang Mai 50200, Thailand; 4 Cluster of Agro Bio-Circular-Green Industry, Faculty of Agro-Industry, Chiang Mai University, Chiang Mai 50100, Thailand; 5 School of Agro-Industry, Faculty of Agro-Industry, Chiang Mai University, Chiang Mai 50100, Thailand; 6 Lanna Rice Research Center, Chiang Mai University, Chiang Mai 50200, Thailand; 7 Department of Animal and Aquatic Sciences, Faculty of Agriculture, Chiang Mai University, Chiang Mai 50200, Thailand; 8 Department of Preventive Medicine and Public Health, Food Science, Toxicology and Forensic Medicine, Faculty of Pharmacy, Universitat de València, 46100 València, Spain

**Keywords:** anthocyanin, antioxidant, collagen stimulator, matrix metalloproteinase 2 inhibition, melanogenesis inhibitor, *Oryza sativa*, rice bran, rice husk, skin whitening

## Abstract

*Oryza sativa* L. cv. Pieisu 1 CMU (PES1CMU) has a high anthocyanin content in the colored bran and high phenolic content in the husk. Biologically active compounds in plants are available as dietary supplements and cosmetics. To expand the utilization of natural resources, PES1CMU will be a natural remedy for skin hyperpigmentation and aging. Cell-free tyrosinase inhibition and scavenging assays were used to screen all extracts, including PES1CMU-rice bran oil (RBO), PES1CMU-defatted rice bran (DFRB), and PES1CMU-husk (H). PES1CMU extracts were first examined in IBMX-stimulated B16 cells and H_2_O_2_-induced fibroblasts. The results exhibited that PES1CMU-DFRB was the most effective inhibitor of mushroom tyrosinase, intracellular melanin production (fold change of 1.11 ± 0.01), and tyrosinase activity (fold change of 1.22 ± 0.10) in IBMX-stimulated B16 cells. Particularly, PES1CMU-DFRB showed a comparable whitening effect to the standard arbutin with no significant difference (*p* > 0.05). Moreover, PES1CMU-DFRB and PES1CMU-H demonstrated strong scavenging activities. After accelerated cell aging caused by H_2_O_2_ exposure in fibroblasts, the levels of malondialdehyde production in all PES1CMU-treated fibroblasts were comparable with those of standard l-ascorbic acid (*p* > 0.05). Besides, PES1CMU-DFRB and PES1CMU-H treatment significantly inhibited collagen degradation against MMP-2 compared to l-ascorbic acid-treated cells (*p* > 0.05). PES1CMU rice-processing wastes (DFRB and H) could become potential natural sources for dermatocosmetic constituents in skin anti-aging and whitening products.

## 1. Introduction

Asia is a well-known rice-growing region with high rice consumption. Additionally, Thailand’s rice production dominates the global rice supply chain [1]. According to sustainable development goals (SDGs), the concept of ‘Responsible Consumption and Production’ focuses on the management of food loss and wasted food [2]. Normally, rice husks are used as animal feed and construction materials [3]. Rice bran is the main source of γ-oryzanol and tocopherols. Rice bran oil has medicinal and health benefits such as cancer prevention, anti-inflammation, and lowering of blood pressure [4]. In line with the results of our previous study, rice bran and husk extracts could be applied as an anti-androgen agent for baldness [5,6].

Pieisu 1 CMU (PES1CMU) is one of the inbred rice cultivars that originated in the north of Thailand. PES1CMU has already received legislative protection as of November 2020. Our previous research found that the rice bran of PES1CMU, purple glutinous rice, was the source of anthocyanins, including cyanidin-3,5-diglucoside and peonidin-3-glucoside [7], which play a vital role in skin whitening effects and anti-aging properties [8,9]. Although, the efficacy of PES1CMU against aesthetic problems has not been evaluated.

In 2027, the global market for skin-whitening and anti-aging products is expected to reach 12.3 [10] and 83.2 billion USD [11], consecutively. In addition, plant resources provide impressive anti-melanogenesis activities and antioxidant properties [9]. Skin aging is associated with a lack of moisture, reduced skin elasticity, age spots, and wrinkle formation. Both intrinsic factors and external stressors remarkably influence skin function. The accumulation of damage from the environment (e.g., pollutants or sunlight) can contribute to the dysfunction of skin epidermal and dermal cells [12]. As a consequence, melanocytes overproduce melanin pigments, and fibroblasts diminish the ability to synthesize collagen due to the increased levels of matrix metalloproteinases (MMPs) [13]. Melanin overproduction in human skin explicitly influences skin dullness, melasma, freckles, and solar lentigines [14]. Likewise, wrinkles are apparently recognized as signs of skin aging. Reactive oxygen species (ROS) impact telomere shortening, cell membrane deconstruction, and mitochondrial impairment in fibroblasts, leading to cell aging. In addition, MMP activation can cause collagen depletion, resulting in skin wrinkling [15].

Considering all of the above, this study aimed to determine the cosmetic potential of the Thai rice cultivar, PES1CMU, in addition to exploring natural resources for skin whitening and anti-aging agents. The PES1CMU was prepared as three distinct extracts (including rice bran oil, defatted rice bran, and rice husk extracts) and examined for their inhibitory effects on melanin production using cell-free mushroom tyrosinase activity and 3-isobutyl-1-methylxanthine (IBMX)-stimulated B16 cells. The antioxidant capacity of PES1CMU was assessed through scavenging assays, metal chelation, and the production of malondialdehyde (MDA) in fibroblasts exposed to hydrogen peroxide (H_2_O_2_). Then, the supernatant of treated fibroblasts was evaluated for collagen-stimulating effect via MMP-2 inhibition.

## 2. Results

### 2.1. Extract Preparations

The extraction yields of rice bran oil (RBO) from the screw press and dichloromethane extraction based on dry weight were 8.82% and 6.11%, respectively. The physical appearance of RBO extract was a homogenous brown-black oil. The crude extract of defatted rice bran was a greasy, dark purple paste. In the case of rice husk, the sample was a dark brown, sticky, and coarse paste. The yields obtained from the maceration of defatted rice bran (DFRB) and rice husk (H) were 9.36% and 2.44% based on dry weight, respectively.

### 2.2. Cytotoxicity Effect

**Figure 1:**
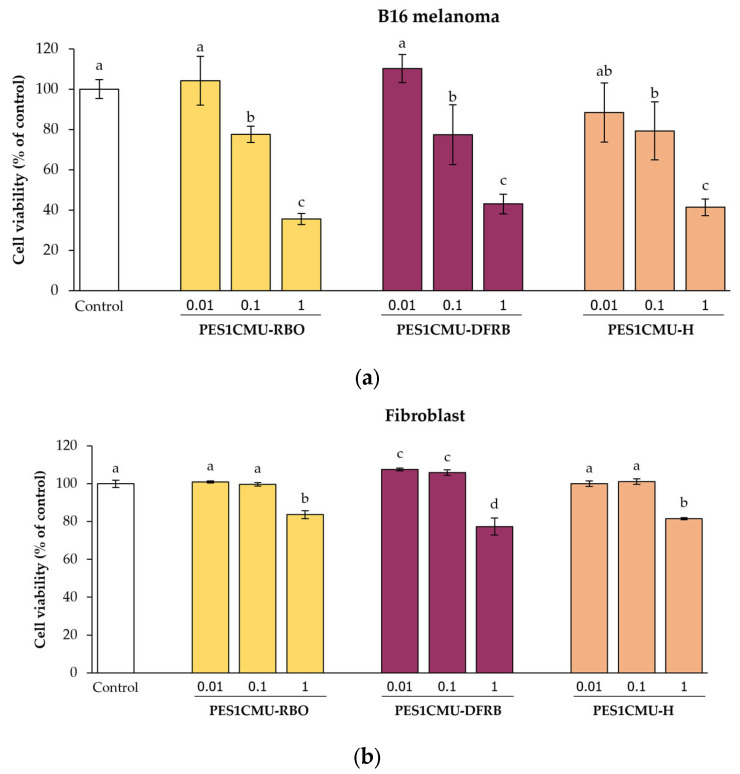
Effects of rice bran oil (PES1CMU−RBO), defatted rice bran extract (PES1CMU−DFRB), and husk extract (PES1CMU−H) of *Oryza sativa* L. cv. PES1CMU on the viability of (**a**) melanoma and (**b**) fibroblast cells exposed to 0.01, 0.1, and 1 mg/mL of extracts after 48 h of incubation. The results were expressed as a percentage of cell viability relative to the untreated control. Data were analyzed using one-way analysis of variance (ANOVA), followed by LSD’s post hoc test. Different letters within each cell treatment indicate a significant difference (*p* < 0.05).

The cytotoxicity effects of PES1CMU extracts on B16 melanoma and fibroblast cells were performed by the sulforhodamine B (SRB) assay to obtain the optimal concentration of extracts. The SRB colorimetric method is used for cytotoxic screening based on protein content in cell lines. The maximum concentration of each extract with low cytotoxicity (>80% cell viability) was used in the following experiments [16]. Melanoma and fibroblast cells were treated for 48 h with the indicated concentrations of PES1CMU-RBO, PES1CMU-DFRB, and PES1CMU-H (0.01, 0.1, and 1 mg/mL). The cell viability of extracts in each cell line was illustrated in Figure 1. All PES1CMU extracts reduced the cell viability of all cell lines in a concentration-dependent manner. After exposure to 0.01 mg/mL of all PES1CMU extracts, no significant cytotoxic effect was observed on B16 melanoma cells. However, the viability of B16 cells was decreased to approximately 78, 78 and 79% of the control after exposure to PES1CMU-RBO, PES1CMU-DFRB, and PES1CMU-H at a concentration of 0.1 mg/mL, respectively (Figure 1a). At the same concentration, PES1CMU-RBO, PES1CMU-DFRB, and PES1CMU-H showed less cytotoxicity on fibroblast cells with the cell viability of 100, 106 and 101% of control, approximately (Figure 1b). Our findings exhibited a concentration-dependent reduction in cell viability after exposure to all PES1CMU extracts, indicating that the higher concentrations of all extracts provoked more cell toxicity. The appropriate concentration of extracts should be considered. Thus, PES1CMU extracts at concentrations up to 0.01 and 0.1 mg/mL were used for subsequent cell-based tests on B16 melanoma and fibroblast cells, respectively.

### 2.3. Whitening Effects

#### 2.3.1. Mushroom Tyrosinase Activity

The preliminary observation of the whitening effect was determined using the mushroom tyrosinase enzyme. The inhibition of mushroom tyrosinase-catalyzed oxidation of monophenolase substrate (l-tyrosine) and diphenolase substrate (l-dihydroxyphenylalanine: l-DOPA) was exhibited in Table 1. PES1CMU-DFRB possessed the most potent inhibitory effect on mushroom tyrosinase activity among all fractions. The IC_50_ values of PES1CMU-DFRB were 0.99 ± 0.30 mg/mL for monophenolase activity and 1.92 ± 0.71 mg/mL for diphenolase activity, which were comparable to the standard arbutin with no significant difference (*p* > 0.05). These were consistent with previous studies on the melanogenesis inhibition of *Andropogon virginicus* and *Dendrobium tosaense*, which demonstrated IC_50_ values of 2.58 mg/mL [17] and 6.40 ± 0.30 mg/mL [18] on the monophenolase and diphenolase activities of mushroom tyrosinase, respectively.

#### 2.3.2. Intracellular Melanin Content

**Figure 2:**
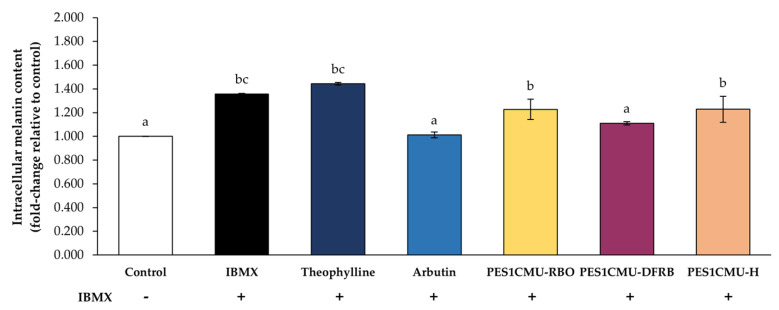
Effects of rice bran oil (PES1CMU−RBO), defatted rice bran extract (PES1CMU−DFRB), and husk extract (PES1CMU−H) of *Oryza sativa* L. cv. PES1CMU on intracellular melanin content in B16 melanoma cells induced with 50 µM IBMX for 48 h. Cells were treated with theophylline, arbutin, or PES1CMU extracts at a concentration of 0.01 mg/mL. Data were analyzed using one−way analysis of variance (ANOVA), followed by LSD’s post hoc test. Different letters within each treatment indicate a significant difference (*p* < 0.05).

In this study, the whitening effect of PES1CMU extracts was further determined through intracellular melanin production and cell-based tyrosinase assay. B16 melanoma cells were stimulated with the cyclic adenosine monophosphate (cAMP) elevator, 50 µM IBMX. IBMX is known to considerably up-regulate melanin production through the cAMP cascade [19]. Theophylline, arbutin, or PES1CMU extracts at 0.01 mg/mL were selected to compare anti-melanogenesis effects on cell-based models. After 48 h of treatment, intracellular melanin was dissolved in an alkaline solution and then measured using the spectrophotometric method, as shown in Figure 2. The results were expressed as a fold change in melanin content compared to the untreated control. IBMX and theophylline treatments can induce melanin production with no significant difference (*p* > 0.05). The intracellular melanin content assay revealed a significant reduction in melanin content in the PES1CMU-DFRB-treated group (fold change of 1.11 ± 0.01) compared to the IBMX-induced group (fold change of 1.36 ± 0.01) and negative control, theophylline-treated group (fold change of 1.44 ± 0.01), respectively. Furthermore, the level of intracellular melanin in PES1CMU-DFRB-treated cells was lower than in the theophylline-treated group at 0.33 ± 0.01-fold change. Melanin content in B16 cells after PES1CMU-DFRB treatment was nearly 1.09 times higher than the arbutin treatment. Interestingly, the effect of PES1CMU-DFRB on melanin production was not significantly different from that of the whitening positive control, arbutin (fold change of 1.01 ± 0.03).

#### 2.3.3. Intracellular Tyrosinase Activity

**Figure 3:**
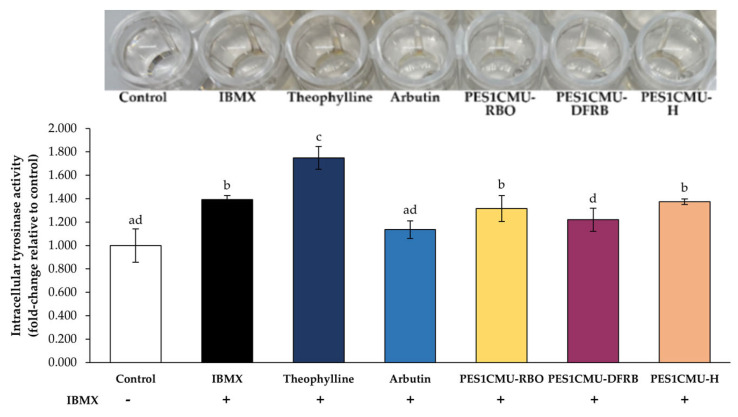
Effects of rice bran oil (PES1CMU−RBO), defatted rice bran extract (PES1CMU−DFRB), and husk extract (PES1CMU−H) of *Oryza sativa* L. cv. PES1CMU extracts on intracellular tyrosinase activity in B16 melanoma cells induced with 50 µM IBMX for 48 h. Cells were treated with theophylline, arbutin, or PES1CMU extracts at a concentration of 0.01 mg/mL. Data were analyzed using one−way analysis of variance (ANOVA), followed by LSD’s post hoc test. Different letters within each treatment indicate a significant difference (*p* < 0.05).

Tyrosinase, a copper-containing metalloenzyme, is an attractive target for melanogenesis inhibitors. The results of the cell-based tyrosinase assay further supported the inhibitory effect on tyrosinase activity (Figure 3). The results were expressed as a fold change in intracellular tyrosinase activity compared to the untreated control. In this study, the tyrosinase activity of B16 cells exposed to IBMX alone (fold change of 1.40 ± 0.03) was significantly elevated compared to untreated cells. In particular, tyrosinase activity was increased to a fold change of 1.75 ± 0.03 in B16 cells treated with standard theophylline. PES1CMU-DFRB showed higher tyrosinase inhibition than the negative control, theophylline-treated group by about 1.43 times. Conversely, the standard arbutin (positive control) suppressed the tyrosinase activity of B16 cells (fold change of 1.14 ± 0.08) at a similar level to PES1CMU-DFRB (fold change of 1.22 ± 0.10) with no significant difference (*p* > 0.05). Intracellular tyrosinase activity after PES1CMU-DFRB treatment was about 1.07 times higher than that of standard arbutin.

### 2.4. Antioxidant Properties

#### 2.4.1. Screening of Antioxidant Activities

Both the 2,2-diphenyl-1-picrylhydrazyl (DPPH) and 2,2′-azino-bis (3-ethylbenzthiazoline-6-sulfonic acid) (ABTS) methods are regularly adopted for the free radical scavenging assays of plant extracts. The antioxidant capacity of DPPH radicals was recognized by the reduction of violet color. Due to pigments in plant extracts, the absorbance reading of DPPH could be affected and variable [20]. The application of the ABTS assay was widely evaluated for hydrophilic and lipophilic antioxidants. Although, ABTS cations may not even be represented as biological free radicals [21]. For the metal chelating assay, the complex formation between the metal and its substrate, such as ferrozine, is measured using the spectroscopic method. Excess free irons and radicals in biological systems implicate the induction of oxidation at lipid components of the plasma membrane [22]. Thus, all three approaches are used concurrently to confirm the antioxidant activities.

In this study, PES1CMU-DFRB showed the highest in vitro antioxidant capacity by the DPPH and ABTS methods (648.39 ± 8.99 and 377.49 ± 19.43 mg TEAC/g extract, respectively), as shown in Table 2. The strongest value for iron chelation was 131.55 ± 19.43 mg EECC/g extract in PES1CMU-H. All extracts were further investigated in a cell-based assay to ensure their antioxidant effects.

#### 2.4.2. Malondialdehyde Production

**Figure 4:**
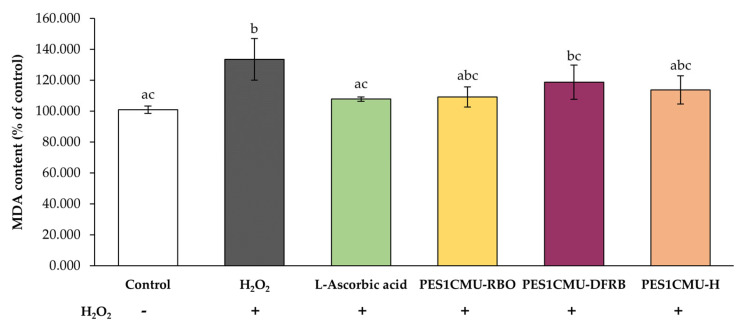
Effects of rice bran oil (PES1CMU−RBO), defatted rice bran extract (PES1CMU−DFRB), and husk extract (PES1CMU−H) of *Oryza sativa* L. cv. PES1CMU extracts on MDA content in fibroblast cells stimulated with 250 µM H_2_O_2_ for 48 h. Cells were treated with l−ascorbic acid or PES1CMU extracts at a concentration of 0.1 mg/mL. The results were expressed as a percentage of MDA content relative to the untreated control. Data were analyzed using one−way analysis of variance (ANOVA), followed by LSD’s post hoc test. Different letters within each treatment indicate a significant difference (*p* < 0.05).

MDA is one of the secondary metabolites from the lipid peroxidation process [23]. The coupling of MDA in biological samples with thiobarbituric acid-reactive substances (TBARS) generates MDA-TBA adducts, which can be detected using spectrophotometry. As shown in Figure 4, MDA levels in fibroblasts increased significantly after H_2_O_2_ exposure to 133.47 ± 13.51% of control (*p* < 0.05), which may cause fibroblast cell necrosis, apoptosis, and skin aging. On the contrary, the MDA content after the pretreatment with the standard antioxidant, l-ascorbic acid (107.80 ± 1.40% of control) was nearly equal to that of untreated cells (100.92 ± 2.43% of control). All PES1CMU extracts, including PES1CMU-RBO, PES1CMU-DFRB, and PES1CMU-H, slightly decreased MDA production after H_2_O_2_ stimulation. On the other hand, the effects of all PES1CMU extracts against lipid peroxidation via MDA production showed no significant difference compared to l-ascorbic acid treatment (*p* > 0.05).

### 2.5. Collagen-Stimulating Effect via MMP-2 Inhibition

**Figure 5:**
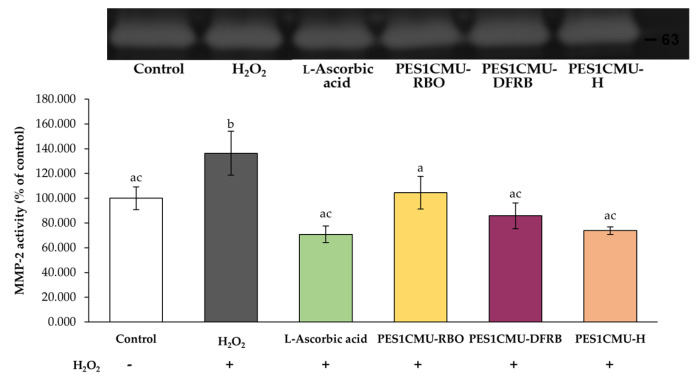
Effects of rice bran oil (PES1CMU−RBO), defatted rice bran extract (PES1CMU−DFRB), and husk extract (PES1CMU−H) of *Oryza sativa* L. cv. PES1CMU extracts on MMP−2 expression in fibroblast cells stimulated with 250 µM H_2_O_2_ for 48 h. Cells were treated with l−ascorbic acid or PES1CMU extracts at a concentration of 0.1 mg/mL. The results were expressed as a percentage of MMP−2 activity relative to the untreated control. Data were analyzed using one−way analysis of variance (ANOVA), followed by LSD’s post hoc test. Different letters within each treatment indicate a significant difference (*p* < 0.05).

Gelatinase A, or MMP-2, is one mediator of type IV collagen degradation which is largely expressed in fibroblast cells. MMP-2 activity can be detected using gelatin zymography. The molecular size of the MMP-2 protein is approximately 63 kDa, which corresponds to active MMP-2 [24,25]. A better collagen stimulator contributes to higher MMP-2 inhibitory capacity. As shown in Figure 5, all PES1CMU extracts illustrated a significant decrease in MMP-2 activity after induction with H_2_O_2_ (*p* < 0.05). The effects of PES1CMU-RBO, PES1CMU-DFRB, PES1CMU-H, and l-ascorbic acid resulted in the reduction of MMP-2 activity to 104.47 ± 13.30, 85.80 ± 10.40, 73.78 ± 3.07, and 70.84 ± 6.81% of control, respectively. Interestingly, there was no significant influence on MMP-2 activity between PES1CMU-DFRB, PES1CMU-H, and l-ascorbic acid. Hence, PES1CMU-DFRB and PES1CMU-H showed comparable collagen-stimulating effects to the standard l-ascorbic acid.

## 3. Discussion

In our previous study, the phenolic contents of PES1CMU bran and husk extracts were found to be high. Particularly, the dominant constituent of PES1CMU bran was anthocyanins, namely cyanidin-3,5-diglucoside and peonidin-3-glucoside [7]. Anthocyanins are mainly found in plant pigments, especially in berry species. The hydroxyl groups in the molecules can contribute to the antioxidant activity against ROS and possess skin protective effects together with a UV-filtering role, skin whitening effect, and anti-aging properties [8,9]. The fatty acid profiles revealed that rice bran was rich in saturated and unsaturated fatty acids [26,27]. In addition, quercetin, phytic acid, and chlorogenic acid were major phenolic compounds in the rice bran portion [7]. In our recent research, the phenolic profiles of rice husk from PES1CMU were abundant in phytic acid, catechin, *o*-coumaric acid, epigallocatechin gallate, ferulic acid, and quercetin, respectively. Surprisingly, the high quercetin content of PES1CMU husk may have the greatest antioxidant effect among other local rice cultivars [28]. In addition, PES1CMU also had the highest δ-tocopherol content of eleven local rice cultivars in rice bran oil [7]. Therefore, PES1CMU extracts were first tested using cell-free experiments, including a mushroom tyrosinase inhibition assay and antioxidant screening methods. After that, the cytotoxic effects of all extracts were established before a subsequent confirmation by IBMX-induced B16 and H_2_O_2_-stimulated fibroblasts.

As mentioned, effective whitening agents should have promising mushroom tyrosinase inhibition, reducing intracellular melanin production and tyrosinase activity in B16 cells. The unpigmented precursor cells, namely melanoblasts, which colonize hair follicles and the skin epidermis, can differentiate into mature melanocytes [29]. The initial substrate in the melanin synthesis, l-tyrosine, is converted by tyrosinase into l-DOPA and l-dopaquinone, respectively. The tyrosinase enzyme is recognized as responsible for melanin synthesis. This enzyme is the main target of promising skin-whitening agents. Commercial skin-whitening agents act as tyrosinase inhibitors, such as hydroquinone, kojic acid, arbutin, and l-ascorbic acid. The first stage-mediated tyrosinase enzyme is the rate-limiting step in the biosynthesis of melanin. Subsequently, l-dopaquinone is further oxidized to l-dopachrome and then produced melanin pigments [30]. Two catalytic stages of tyrosinase involve monophenolase and diphenolase reactions. l-tyrosine and l-DOPA are substrates for monophenolase and diphenolase activities, respectively. The molecular docking analysis reported that tyrosinase interacts with the hydroxyl group of the l-tyrosine substrate towards copper (Cu^2+^) B atom (distance 1.97 Å) and histidine residue (His208) using a π–π stacking interaction. The phenol group of l-DOPA is positioned towards a Cu^2+^B atom (distance 2.87 Å) for the l-DOPA substrate [31]. In the present study, PES1CMU-DFRB possessed impressive tyrosinase inhibition against the mushroom tyrosinase enzyme, with IC_50_ values of 0.99 ± 0.30 and 1.92 ± 0.71 mg/mL for monophenolase and diphenolase activities, respectively. Nonetheless, the inhibition of mushroom tyrosinase might not correlate with the effect on mammalian tyrosinase [32]. To illustrate, the IC_50_ values α-arbutin and β-arbutin were 6499 ± 137 and 1687 ± 181 μM, respectively, for monophenolase activity. Conversely, α-arbutin and β-arbutin at a concentration of 43.8–700 µM showed significant inhibitory effects against B16 tyrosinase activity [33]. Thus, the mammalian cell-based model, such as B16 melanoma, was selected for screening melanogenesis. In this study, PES1CMU-DFRB significantly down-regulated the melanin content and tyrosinase activity in IBMX-induced B16 cells. These findings confirmed that the whitening effect of PES1CMU-DFRB may be due to the presence of cyanidin-3,5-diglucoside (650.55 ± 1.65 mg/100 g rice bran) [7]. Whitening effects were found in other natural plants with anthocyanins, such as *Vitis vinifera* [34], *Hibiscus syriacus* [35], and *Pistacia vera* [36]. In consequence, kinetic studies revealed that cyanidin-3-*O*-glucoside acts as a competitive inhibitor for tyrosinase, with an inhibition constant of 40.31 ± 3.61 µM. In addition, molecular simulations demonstrated that the oxygen atom at ring A of cyanidin-3-*O*-glucoside can interact with the central Cu^2+^ of the human tyrosinase enzyme with the lowest binding energy of −10.8 kcal/mol [37]. Although Lee et al. reported that cyanidin-3-*O*-glucoside slightly reduced the activity of murine tyrosinase enzyme by comparison to arbutin as a positive control [38]. In this study, biologically active compounds that enrich PES1CMU-DFRB can decrease the tyrosinase activity and melanin production in B16 cells compared with the standard arbutin. Moreover, the metal ion chelating activity of PES1CMU-DFRB could synergize the Cu^2+^ binding effect of the tyrosinase enzyme. However, the deep mechanism via gene, protein expression and molecular verification methods, such as qPCR and Western blot, should be investigated further to confirm our speculation.

Natural bioactive compounds with strong scavenging abilities and the ability to prevent lipid peroxidation are suitable as anti-aging agents for cosmetics. The mechanisms of antioxidant defense and redox balance markedly regulate cell homeostasis. In the mitochondria, the main ROS are formed as H_2_O_2_. An excess of H_2_O_2_ can influence either necrosis or apoptosis of epidermal skin cells [39]. The hydroxyl radicals from H_2_O_2_ can bind to membrane lipids, leading to the excessive production of reactive lipid species (RLS) and an imbalance in a redox system, respectively. Lipid peroxidation is a consequence of the oxidative stress response in the biological system [40]. Polyunsaturated fatty acids, part of the lipid membrane, are the target site of hydroxyl radical-derived oxidative stress. During the autoxidation of phospholipid bilayers, reduced forms of iron atoms are used to generate reactive radicals via the Fenton reaction [41]. The end products of lipid peroxidation include 4-hydroxy-2-nonenal (HNE), malondialdehyde (MDA), and acrolein, which have been linked to aging disorders, cell apoptosis, and pro-inflammatory response [42]. Additionally, impaired membrane homeostasis leads to cell death, or ferroptosis [43]. In this study, PES1CMU-DFRB enhanced scavenging capacity against DPPH and ABTS radicals. Moreover, all PES1CMU extracts showed a reduction of MDA levels in fibroblasts after H_2_O_2_ exposure at a comparable level to l-ascorbic acid-treated cells (*p >* 0.05). Due to the unsaturated free fatty acids of PES1CMU rice bran, the contents of oleic acid and linoleic acid were about 40 g and 36 g per 100 g of crude fat, respectively [7]. Scientific evidence reported that oleic acid had a protective effect on antioxidant enzymes [44] as well as inhibitory effects on heavy ion-induced cell damage and ferroptosis through lipid peroxidation [45]. Previous experiments supported that oleic acid could decline the amount of ROS through the protection of the active sites of antioxidant enzymes and free radical quenching after cadmium-stimulated cell injury [46]. Similarly, plant extracts, including *Cannabis sativa* [47] and *Olea europaea* [48], had antioxidant properties due to the presence of oleic acid. Each 100 g of PES1CMU bran contained 1.44 ± 0.00 mg of quercetin, 1.19 ± 0.00 mg of phytic acid, and 0.93 ± 0.02 mg of chlorogenic acid, respectively [7], resulting in a decreasing MDA level. Previous research suggested that the administration of quercetin to mice can protect against oxidative stress that results from ferrous sulfate-induced organ injury [49]. Additionally, other studies confirmed that quercetin, a biologically active phenolic compound, can also alleviate the production of intracellular H_2_O_2_ and the lipid peroxidation process in fibroblasts after UV radiation [50]. This was consistent with other quercetin-enriched plants such as *Prunus pseudocerasus* [51] and *Moringa oleifera* [52], which provide antioxidant properties against H_2_O_2_ induction. Thus, the antioxidant effect of PES1CMU-DFRB might be from the contents of oleic acid and quercetin in the rice barn of PES1CMU.

Collagen in the skin acts as a major supportive structure. The degradation of collagen in the skin, particularly on the face, is an important factor in skin aging that begins around the age of 30 [53]. As known, collagen is composed of triple helices of different amino acid compositions. Alterations in the metabolism of collagen affect the physiology of the skin and also lead to skin aging. Type IV collagen plays an essential role in controlling cutaneous transepidermal water loss [54] and reducing skin roughness and wrinkles [55]. Fibroblast cells maintain the extracellular matrix (ECM) networks by producing collagen, elastin, and glycosaminoglycans [56]. MMPs, a group of zinc-binding enzymes, play a vital role in the breakdown of ECM. Particularly, gelatinases, including MMP-2 and MMP-9, can cleave the structure of collagens and proteoglycans, which influence skin strength. Therefore, the biosynthesis of ECM or the reduction of MMP enzymes in fibroblasts is the primary target for anti-aging agents. For instance, l-ascorbic acid is an essential physiological cofactor for the hydroxylation of procollagen proline and lysine [57]. Furthermore, oral administration of collagen peptides can decrease the degradation of collagen and support skin health characteristics such as moisture and elasticity [58]. Rabelo et al. indicated that ascorbic acid used to be the standard compound against the MMP-2 enzyme [59]. In the present study, PES1CMU-DFRB could downregulate the expression of the MMP-2 enzyme in H_2_O_2_-stimulated fibroblasts at the comparable level to the standard collagen stimulator, l-ascorbic acid, with no significant difference (*p* > 0.05). In addition, published studies revealed the inhibitory effect of quercetin on radical production and the expression of MMP [60]. Along with previous works, quercetin could reduce the expression of MMP-2 based on the suppression of nuclear factor-kB (NF-kB) [61] or modulation of superoxide dismutase (SOD) activity [62]. Several studies demonstrated that the quercetin compound found in plant extracts like *Oenothera biennis* [63] and *Magnolia officinalis* [64] can inhibit MMP-2 secretion and be used as an anti-aging agent. The inhibition of MMP-2 activity influences a decrease in the destruction of collagen in the skin. Therefore, the source of quercetin in PES1CMU-DFRB could inhibit the destruction of collagen against MMP-2.

## 4. Materials and Methods

### 4.1. Extraction Method

**Figure 6:**
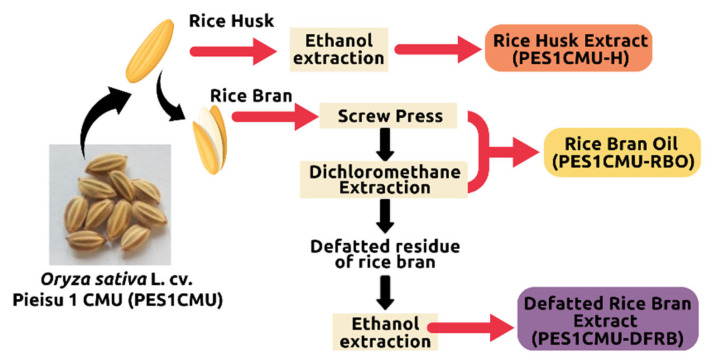
An overview of the extraction process of *Oryza sativa* L. cv. Pieisu 1 CMU.

The Thai rice variety ‘Pieisu 1 CMU’ was collected by the Lanna Rice Research Center, Chiang Mai University, Chiang Mai, Thailand in October 2021. The extraction process (Figure 6) was performed by the Pharmaceutical and Natural Products Research and Development Unit (PNPRDU), Chiang Mai University, Chiang Mai, Thailand. Firstly, the rice husk (100 g) was separated and soaked in a 95% ethanol solution (6 L). After 48 h of maceration, the rice husk extract was concentrated by a rotary evaporator. Secondly, rice bran (1000 g) was removed from the rice grain and compressed using the screw extruder to obtain the rice bran oil. The residue oil content (100 g) was further extracted with dichloromethane (2 L) for 72 h to obtain the rice bran oil. Finally, the ethanol extraction of the de-oiled rice bran (100 g) was carried out to obtain the defatted rice bran extract. The different extracts from Pieisu 1 CMU were labeled as PES1CMU-RBO for the essential oil from the rice bran, PES1CMU-DFRB for the extract from the defatted residue of the rice bran, and PES1CMU-H for the extract from the rice husk.

### 4.2. Cytotoxicity Assay

#### 4.2.1. Cell Culture

The mouse skin melanoma cell line (B16 melanoma; JCRB0202) and immortalized human fibroblast cell line (OUMS-36T-4F; JCRB1006.4F) were obtained from the JCRB Cell Bank (Osaka, Japan). B16 melanoma cells were cultured in Eagle’s minimal essential medium (MEM) (Gibco Life Technologies, Grand Island, NY, USA) containing 10% fetal bovine serum (HyClone^TM^, GE Healthcare Life Sciences Laboratories, South Logan, UT, USA) and 1% penicillin/streptomycin (Capricorn Scientific GmbH, Ebsdorfergrund, Germany). Human fibroblast cells were cultured in Dulbecco’s Modified Eagle Medium (DMEM) (Gibco Life Technologies, Grand Island, NY, USA) containing 10% fetal bovine serum and 1% penicillin/streptomycin. All cells were maintained in a humidified incubator at 37 °C with 5% CO_2_. All experiments were performed with the cells between passages 3 and 10.

#### 4.2.2. SRB Assay

Cell viability was measured by the protein-specific dye sulforhodamine B (SRB) (Sigma Chemical, St. Louis, MO, USA) as previously described [16]. B16 and fibroblast cells were seeded separately into 96-well plates (10^4^ cells/well) and incubated for 24 h. Cells were treated with 0.01–1 mg/mL extracts for an additional 48 h. The extract-containing supernatant was discarded and replaced with 50% trichloroacetic acid (PanReac AppliChem, Barcelona, Spain) to fix cells on plates at 4 °C for 1 h. The 0.04% SRB solution was added to stain the bounded cells at room temperature for 30 min. The unbound dye in each well was removed using a 1% acetic acid solution and allowed to dry overnight. A tris solution (Vivantis, Selangor, Malaysia) was added to solubilize the attached dye in the cells. The absorbance was measured at 515 nm using a microplate reader (EZ2000 Biochrome Ltd., Cambridge, UK). The results were expressed as the percentage of cell viability compared to untreated cells.

### 4.3. Determination of Whitening Effects

#### 4.3.1. Cell-Free Tyrosinase Inhibition

The measurement of mushroom tyrosinase activity was modified from the published method [65,66]. l-Tyrosine (Bio Basic, Ontario, Canada) or l-DOPA (Sigma Chemical, St. Louis, MO, USA) were used as substrates for monophenolase and diphenolase activity, respectively. Each sample was diluted to a series of concentrations (0.039, 0.078, 0.156, 0.3125, 0.625, 1.25, 2.5, and 5 mg/mL). Mushroom tyrosinase (Sigma Chemical, St. Louis, MO, USA) was dissolved in 0.1 M phosphate buffer (pH = 6.8) to obtain the final concentration of 100 units/mL. The reaction was started by adding 40 µL of sample solution, 80 µL of phosphate buffer, 40 µL of mushroom tyrosinase, and 40 µL of substrates (1.5 mM l-tyrosine or 2.5 mM l-DOPA solution). After incubation at room temperature for 15 min, the l-dopachrome formation was measured at 475 nm. The percentage of mushroom tyrosinase inhibition was calculated using the following equation:

The inhibition of mushroom tyrosinase activity = [(A − B) – (C − D)]/(A − B) × 100,(1)

where A = vehicle control, B: = vehicle control without mushroom tyrosinase, C = sample mixed with mushroom tyrosinase, and D = sample without mushroom tyrosinase. The results were expressed as IC_50_ values (the concentration that caused 50% mushroom tyrosinase inhibition).

#### 4.3.2. Cellular Melanin Content Assay

The intracellular melanin levels were determined as previously described [19,67] with some modifications. B16 cells were seeded in 6-well plates at a concentration of 2.5 × 10^5^ cells/well and allowed to attach for 24 h. This assay was divided into 7 groups as follows: control, non-treatment; IBMX: 50 µM IBMX (PanReac AppliChem, Barcelona, Spain); theophylline: IBMX + 0.01 mg/mL theophylline (Sigma Chemical, St. Louis, MO, USA); arbutin: IBMX + 0.01 mg/mL arbutin (Sigma Chemical, St. Louis, MO, USA); PES1CMU-RBO: IBMX + 0.01 mg/mL rice bran oil; PES1CMU-DFRB: IBMX + 0.01 mg/mL de-oiled rice bran extract; and PES1CMU-H: IBMX + 0.01 mg/mL husk extract. After 48 h of incubation, cell pellets were collected and lysed with 1 N NaOH containing 10% DMSO at 80 °C for 30 min. The intracellular melanin release was measured at 405 nm using a microplate reader. The results were expressed as a fold change in melanin content compared to the control.

#### 4.3.3. Cellular Tyrosinase Assay

Cellular tyrosinase activity was conducted according to the previous method [19,67] with a slight modification. Briefly, B16 cells were incubated with samples and co-treated with 50 µM IBMX for 48 h. Cells were harvested and lysed with a PBS solution containing 1% Triton X-100 (VWR Life Science, Solon, OH, USA) at −20 °C for 30 min. Then, cell lysates were ruptured using the vortex mixer and further clarified by centrifugation at 11,000× *g* for 10 min. The supernatant was obtained to react with 5 mM l-DOPA at 37 °C for 1 h. The l-dopachrome formation was measured at 475 nm using a microplate reader. The results were expressed as a fold change in tyrosinase activity compared to the control.

### 4.4. Determination of Antioxidant Properties

#### 4.4.1. DPPH Scavenging Assay

The scavenging methods were performed on DPPH assay, ABTS assay, and metal chelation as previously described [6]. Briefly, 100 µL of each sample was allowed to mix with 50 µL of DPPH solution (Sigma Chemical, St. Louis, MO, USA) in a 96-well plate and incubated in the dark at room temperature. After 30 min of incubation, the DPPH radicals were detected spectrophotometrically at 515 nm. The percentage of scavenging activity against DPPH radicals was calculated using the following equation:

DPPH radical scavenging activity = [(A − B) − (C − D)]/(A − B) × 100,(2)

where A = DPPH radicals, B: = vehicle control, C = sample mixed with DPPH radicals, and D = sample without DPPH radicals. The results were expressed as TEAC values (mg Trolox/g extract).

#### 4.4.2. ABTS Scavenging Assay

The sample solution (25 µL) and ABTS working solution (200 µL) (Sigma Chemical, St. Louis, MO, USA) were reacted at room temperature for around 10 min. The optimal densities of ABTS radicals were determined at 734 nm. The percentage of scavenging activity against ABTS radicals was calculated using the following equation:

ABTS radical scavenging activity = [(A − B) − (C − D)]/(A − B) × 100,(3)

where A = ABTS radicals, B: = vehicle control, C = sample mixed with ABTS radicals, D = sample without ABTS radicals. The results were expressed as TEAC values (mg Trolox/g extract).

#### 4.4.3. Iron Chelating Assay

The reagents were prepared from 3-(2-yyridyl)-5,6-diphenyl-1,2,4-triazine-4′,4′′-disulfonic acid sodium salt (ferrozine) and iron (II) chloride tetrahydrate (FeCl_2_ · 4H_2_O) (Sigma Chemical, St. Louis, MO, USA). The sample (100 µL) was mixed with 50 µL of 5 mM Ferrozine. Afterward, 2 mM FeCl_2_ was added into each well and incubated for 30 min. The absorbance of the ferrous-ferrozine complex was measured at 562 nm. The percentage of iron chelating activity was calculated using the following equation:

Iron chelating activity = [(A − B) − (C − D)]/(A − B) × 100,(4)

where A = ferrous-ferrozine complex, B: = vehicle control, C = sample mixed with ferrous and ferrozine, and D = sample without ferrozine. The results were expressed as EECC values (mg EDTA/g extract).

#### 4.4.4. Thiobarbituric Acid Reactive Substances (TBARS) Method

The measurement of MDA content was adapted from previous works [68,69]. Fibroblast cells were placed into 6-well plates at a concentration of 2.5 × 10^5^ cells/well and incubated for 24 h. The medium was replaced with samples. After 24 h, cells were induced with 0.2 mM H_2_O_2_ for an additional 24 h. The cell lysates were harvested to react with 0.6% TBA solution (BDH Chem. Ltd., Poole, UK) at 100 °C for 10 min. The reaction mixture was then cooled at −20 °C for 5 min to stop the reaction. The chromogen MDA-TBA was measured at 532 nm. The results were expressed as the percentage of MDA content compared to untreated fibroblasts.

### 4.5. MMP-2 Inhibitory Activity by Gelatin Zymography

The expression of MMP-2 in fibroblast cells was performed by the gelatin zymogram as previously published [70]. The supernatant was cleared by centrifugation at 3000× *g* for 3 min and loaded onto 10% acrylamide-sodium dodecyl sulfate (SDS) gels containing 0.1% gelatin. After electrophoresis, gels were washed in 2.5% Triton X-100 solution for 20 min and incubated with the developing buffer (50 mM Tris pH = 7.5, 5 mM calcium chloride, and 0.01% sodium azide) at 37 °C for 24 h. Gels were subsequently stained with 0.5% Coomassie brilliant blue R-250 solution (Bio Basic, Ontario, Canada) for 60 min and washed three times in the destaining solution with gentle shaking. The gel images and protein band intensities were detected using the Gel Doc™ EZ System (Version 3.0; Bio-Rad). The percentage of MMP-2 activity was compared to the control.

### 4.6. Statistical Analysis

All experiments were performed in triplicate. Data were represented as the mean ± standard error. Statistical analysis was conducted by SPSS 23.0 software (SPSS Inc., Chicago, IL, USA). The data were subjected to a one-way analysis of variance (ANOVA) with LSD’s post hoc test. Statistical significance was considered as *p* < 0.05.

## 5. Conclusions

In this study, three different extracts from PES1CMU were selected to determine the whitening activity via mushroom tyrosinase and cell-based assays. Furthermore, the antioxidant properties of PES1CMU extracts were performed based on DPPH, ABTS radicals, iron ions, and malondialdehyde production in fibroblasts. The fibroblast supernatant was further examined. MMP-2 inhibitory activity in skin fibroblasts, which is related to collagen production capability, was conducted. Results showed that anthocyanin-rich PES1CMU-DFRB could diminish the activity of the tyrosinase enzyme responsible for a melanogenesis inhibitor as a skin-whitening agent. Moreover, PES1CMU-DFRB illustrated impressive antioxidant capacities against DPPH, ABTS radicals, and malondialdehyde production. Particularly, PES1CMU-DFRB also presented a significant reduction in the gene expression of MMP-2 enzyme protein production. It was strongly suggested that PES1CMU-DFRB could reduce melanin production, protect the lipid membrane of fibroblasts, and decrease the destruction of collagen. These actions were achieved due to the high fatty acid and phenolic contents in the rice bran portion of PES1CMU. PES1CMU-DFRB could be considered a new active ingredient for cosmetic application with potential for skin whitening, antioxidants, and collagen stimulation in a single extract.

## Figures and Tables

**Table 1:** Inhibitory effects of *Oryza sativa* L. cv. PES1CMU extracts on cell-free mushroom tyrosinase activity.

Samples	IC_50_ (mg/mL) Monophenolase Activity	IC_50_ (mg/mL) Diphenolase Activity
PES1CMU−RBO	12.54 ± 0.48 ^a^	23.14 ± 3.60 ^a^
PES1CMU−DFRB	0.99 ± 0.30 ^b^	1.92 ± 0.71 ^b^
PES1CMU−H	2.90 ± 0.04 ^c^	3.46 ± 0.00 ^b^
Standard arbutin	0.51 ± 0.03 ^b^	3.44 ± 0.04 ^b^

PES1CMU−RBO: rice bran oil of *Oryza sativa* L. cv. PES1CMU; PES1CMU−DFRB: defatted rice bran extract of *Oryza sativa* L. cv. PES1CMU; PES1CMU−H: husk extract of *Oryza sativa* L. cv. PES1CMU; IC_50_: the 50% tyrosinase inhibitory concentration. Data were analyzed using one−way analysis of variance (ANOVA), followed by LSD’s post hoc test. Different letters within each experiment indicate a significant difference (*p* < 0.05).

**Table 2:** DPPH radical scavenging assay, ABTS radical scavenging assay, and iron chelation assay of *Oryza sativa* L. cv. PES1CMU extracts.

Samples	DPPH-TEAC (mg/g)	ABTS-TEAC (mg/g)	Iron Chelation-EECC (mg/g)
PES1CMU−RBO	112.98 ± 1.57 ^a^	11.36 ± 0.58 ^a^	90.56 ± 17.70 ^ab^
PES1CMU−DFRB	648.39 ± 8.99 ^b^	377.49 ± 19.43 ^b^	65.61 ± 12.82 ^a^
PES1CMU−H	214.13 ± 2.97 ^c^	192.20 ± 9.89 ^c^	131.55 ± 25.71 ^b^

PES1CMU−RBO: rice bran oil of *Oryza sativa* L. cv. PES1CMU; PES1CMU−DFRB: defatted rice bran extract of *Oryza sativa* L. cv. PES1CMU; PES1CMU−H: husk extract of *Oryza sativa* L. cv. PES1CMU; DPPH: 2,2−diphenyl−1−picrylhydrazyl; ABTS: 2,2′−azino−bis (3−ethylbenzthiazoline−6−sulfonic acid); TEAC: Trolox Equivalent Antioxidant Capacity; EECC: EDTA Equivalent iron Chelation Capacity. Data were analyzed using one−way analysis of variance (ANOVA), followed by LSD’s post hoc test. Different letters within each treatment indicate a significant difference (*p* < 0.05).

## Data Availability

Not applicable.
